# Identification of CD320, SLC44A1 and TNFRSF13B as potential novel therapeutic targets for CAR T-cell therapy in multiple myeloma

**DOI:** 10.3389/fmed.2025.1737919

**Published:** 2026-01-13

**Authors:** Francesca Garofano, Anna Maria Corsale, Marta Biondo, Andrea Romano, Ingo Schmidt-Wolf, Anna Maria Gullà, Sergio Siragusa, Cirino Botta

**Affiliations:** 1 Laboratory of Experimental Hematology and Immunogenomics, University Hospital of Palermo, Department of Health Promotion, Mother and Child Care, Internal Medicine and Medical Specialties, Hematology Unit, University of Palermo, Palermo, Italy; 2 Central Laboratory of Advanced Diagnosis and Biomedical Research CLADiBioR, University Hospital of Palermo, University of Palermo, Palermo, Italy; 3 University Hospital of Catania, University of Catania, Catania, Italy; 4 University Hospital of Palermo, University of Palermo, Palermo, Italy; 5 Department of Integrated Oncology, Center for Integrated Oncology (CIO), University Hospital Bonn, Bonn, Germany; 6 Candiolo Cancer Institute, FPO-IRCCS, Candiolo, Italy

**Keywords:** CAR T-cell therapy, CD320, moleculartargets, multiple myeloma, single-cell RNA sequencing

## Abstract

**Introduction:**

Multiple myeloma (MM) remains incurable despite effective therapies, with most patients eventually relapsing. Chimeric antigen receptor (CAR) T-cell therapy has improved treatment options but is limited by antigen escape and lack of durable responses. To expand the spectrum of actionable antigens, we sought to identify novel CAR-T actionable targets.

**Methods:**

We analyzed 11 publicly available single-cell RNA sequencing datasets from patients with monoclonal gammopathy of undetermined significance (MGUS), smoldering MM (SMM), MM, relapsed/refractory MM (RRMM) and healthy donors. Plasma cell–specific surface proteins were identified via bioinformatic filtering and validated on external bulk transcriptomic database of ~2,000 patients and by flow cytometry on MM cell lines and primary bone marrow samples.

**Results:**

We identified 15 plasma cell–associated surface proteins, including BCMA, CD138, CD38, and SLAMF7. Five molecules—TNFRSF13B (TACI), CD59, FCGR2B (CD32B), SLC44A1 (CD92), and CD320—were prioritized for further study. CD320 and SLC44A1 were upregulated with disease stage and associated with poor survival, while TNFRSF13B, CD59, and FCGR2B were enriched in advanced disease and linked to better outcomes. Cytogenetic clustering linked these molecules to specific genetic backgrounds, suggesting subtyperelated expression patterns. Flow cytometry confirmed the surface expression of CD59, SLC44A1, TNFRSF13B and CD320.

**Discussion:**

CD320, SLC44A1, and TNFRSF13B are promising, clinically relevant targets for CAR T-cell therapy in MM. Their stage-specific expression and prognostic significance support their potential to enhance existing immunotherapeutic strategies.

## 1 Introduction

Multiple Myeloma (MM) is a hematological malignancy characterized by the uncontrolled proliferation of clonal plasma cells (PCs) within the bone marrow. This malignancy poses significant challenges in clinical management, largely due to its complex disease biology and the high rate of relapse experienced by patients (1–3). Despite significant therapeutic advances, including proteasome inhibitors, immunomodulatory drugs, monoclonal antibodies, and autologous stem-cell transplantation, MM remains largely incurable, with most patients experiencing multiple relapses and eventually succumbing to refractory disease (4–11). This clinical reality underscores the urgent need for novel therapeutic strategies that can improve both the depth and durability of response (12–14). MM typically progresses from precursor conditions, namely monoclonal gammopathy of undetermined significance (MGUS) and smoldering multiple myeloma (SMM) (15–17). While these early stages are often asymptomatic, they carry the potential to evolve into full-blown MM, which presents a significant treatment challenge (18, 19). Immunotherapy has transformed the treatment landscape of MM. Chimeric antigen receptor (CAR) T-cell therapies targeting B-cell maturation antigen (BCMA) have demonstrated remarkable efficacy in heavily pretreated patients (1, 7, 9, 11, 13, 14, 20). However, despite these advancements, several critical challenges remain unresolved, including treatment resistance, antigen escape, and severe therapy-related toxicities such as cytokine release syndrome (CRS) and neurotoxicity (21–25). The heterogeneous nature of MM, driven by genetic and molecular diversity within the clonal plasma cell population, complicates the development of universally effective therapies (17, 26–29). This heterogeneity not only contributes to treatment resistance but also necessitates the identification of new, more precise therapeutic targets that can be leveraged to improve the specificity and efficacy of treatments, particularly in the context of CAR T-cell therapy (3, 7, 21, 30). CAR T-cell therapy has emerged as a revolutionary approach, offering the potential for long-term remission in some patients (31). However, the effectiveness of this therapy is often limited by factors such as the loss of targeted antigens on malignant cells, the immunogenicity of the CAR T cells themselves, the durability of the therapeutic response and the exhaustion of T cells (32–36).

Single-cell RNA sequencing (scRNA-seq) has emerged as a powerful tool to dissect the molecular heterogeneity of MM at unprecedented resolution (37). By capturing gene expression profiles at the single-cell level, scRNA-seq enables the identification of cell surface proteins differentially expressed on malignant PCs compared to normal counterparts. This approach is particularly well-suited for discovering novel therapeutic targets, since CAR T-cell design requires the antigen to be expressed on the surface of malignant PCs while minimizing off-target effects on healthy tissues (38, 39).

Recent studies have applied scRNA-seq to MM and its precursor stages, such as monoclonal gammopathy of undetermined significance (MGUS) and smoldering multiple myeloma (SMM), highlighting transcriptional programs underlying disease progression, immune evasion, and treatment resistance. However, comprehensive analyses that integrate scRNA-seq findings across multiple independent datasets and validate candidate targets using both clinical outcome correlations and experimental assays remain limited. Here, we systematically interrogated 11 publicly available scRNA-seq datasets spanning healthy donors (HD), MGUS, SMM, newly diagnosed MM, and relapsed/refractory MM (RRMM). By focusing on genes encoding surface proteins, we identified candidate molecules with differential expression in malignant PCs. To refine their potential clinical relevance, we integrated our findings with bulk transcriptomic datasets comprising approximately 2,000 patients and performed validation through flow cytometry in both MM cell lines and primary patient samples. Our aim was to identify novel, clinically meaningful antigens that could expand the repertoire of CAR T-cell targets and help overcome current limitations in MM immunotherapy.

## 2 Materials and methods

### 2.1 Single-cell RNA sequencing (scRNA-seq) dataset analysis

We analyzed 11 publicly available single-cell RNA sequencing (scRNA-seq) datasets (Gene Expression Omnibus GEO accession numbers: GSE271107, GSE271915, GSE120221, GSE189460, GSE223060, GSE210079, GSE145977, GSE124310, GSE161801, GSE163278, GSE161722), comprising 147 patients with plasma cell disorders (25 MGUS, 14 SMM, 76 MM, 32 RRMM) and 52 healthy donors (HD). Only bone marrow-derived samples were included. Initial quality control (QC) procedures were implemented using Python and the Scanpy package. A uniform mitochondrial content filter was applied across all datasets, retaining cells with mitochondrial gene expression between 1 and 10% of total transcripts. Subsequently, additional dataset-specific filters were applied based on transcriptomic complexity. For each dataset, we computed the total number of detected transcripts and the number of unique expressed genes per cell. A linear relationship in log–log scale was fitted between these two metrics using scikit-learn, and cells with substantial deviation from the regression line were excluded (minimum gene counts >200 genes per cell), maximum transcript counts <30,000 Unique Molecular Identifiers (UMIs) per cell. This two-step QC approach ensured consistent filtering of low-quality cells while adapting to dataset-specific distributions. Post-QC datasets were imported into RStudio, where cell type annotation was conducted using the Seurat framework. As a reference, we employed the SeuratData object “bmcite” (human bone marrow mononuclear cells). Mapping and annotation were performed iteratively, one dataset and one disease condition at a time. Query cells were projected into a precomputed reference embedding, and dimensionality reduction for visualization was achieved by mapping the query data onto the reference Uniform Manifold Approximation and Projection (UMAP) space, rather than recomputing a new embedding. Notably, PCs appeared as a relatively small population and T and NK cell subsets were separated at larger distances. This pattern reflects the mapping onto a reference dataset and is consistent with accurate lineage assignment. Following annotation, we focused on identifying plasma cell-specific marker genes. For each dataset, we applied the tl.rank_genes_groups (method = “wilcoxon”) function in Scanpy, ranking genes based on differential expression between plasma cells and all other annotated cell types. Resulting gene lists from each dataset were intersected to identify consistently upregulated genes across GSE. From this intersection, we retained only genes also annotated as surface markers within the human Cluster of Differentiation (CD) molecule database. In parallel to the Scanpy-based pipeline, we also implemented an independent analysis using the Seurat v5 framework to validate and extend our findings. The results of this approach are summarized in a heatmap provided in the Supplementary Figure 1.

To explore cytogenetic substructure, we generated pseudobulk transcriptomes by randomly sampling 100 PCs per patient (*n* = 54 newly diagnosed MM, excluding RRMM). Expression data were normalized and clustered using hierarchical clustering based on proxies of cytogenetic lesions (FGFR3, CCND1, CCND2, CCND3, MAF, MAFB, MCL1, IL6R).

For external validation, we integrated findings with bulk gene expression datasets from approximately 2,000 MM patients (GSE4204, GSE2658, GSE57317, GSE4581, GSE4452, GSE9782, and CoMMpass NCT01454297). Prognostic relevance was assessed using Kaplan–Meier and Cox proportional hazards models.

### 2.2 Cell cultures

The multiple myeloma cell lines AMO-1, U-266, OPM-2, RPMI8226 and H929 (obtained from American Type Culture Collection ATCC, USA) were cultured in T75 flasks using complete medium (Stable-Cell RPMI-1640 medium with stable glutamine and sodium bicarbonate, provided by Sigma-Aldrich, USA), supplemented with 25 mM N-2-hydroxyethylpiperazine-N′-2-ethanesulfonic acid (HEPES) buffer, 100 IU/mL penicillin, and 0.1 mg/mL streptomycin (from Euroclone, Italy). For the AMO-1 cell line, 20% heat-inactivated fetal bovine serum (from Dominique Dutscher, Issy-les-Moulineaux, France) was used, while for the U-266, OPM-2, RPMI8226 and H929 cell lines, 10% heat-inactivated fetal bovine serum was used. The cultures were maintained in an incubator at 37 °C with 5% CO_2_, with a cell concentration range of 3 × 10^5^–1 × 10^6^ viable cells/mL, and the culture medium was replaced three times a week.

### 2.3 Isolation of bone marrow mononuclear cells (BMMCs)

Bone marrow mononuclear cells (BMMCs) were isolated from bone marrow (BM) samples collected in EDTA tubes from newly diagnosed and untreated MM patients, following approval from the Ethics Committee of the University Hospital of Palermo (n. 05/2021 and 02/2022 MMvision) and acquisition of signed informed consent. Samples were processed within 24 h. The blood was filtered and mixed with Dulbecco’s phosphate-buffered saline (DPBS) (Euroclone, Italy) in a 1:1 ratio in a 50 mL Falcon tube. Subsequently, the mixture was carefully transferred to another Falcon tube containing Ficoll-Paque PLUS (Cytiva, Italy), followed by density gradient centrifugation (1,500 rpm for 10 min). After centrifugation, the collected BMMCs were washed twice with DPBS, followed by erythrocyte lysis using a red blood cell lysis buffer (ammonium chloride), and two additional washes with DPBS in a centrifuge at 1600 rpm for 5 min.

### 2.4 Flow cytometry validation

Flow cytometry was performed on patient-derived BMMCs and MM cell lines to validate candidate antigen expression. All BM samples and MM cell lines were stained according to the manufacturer’s guidelines. Antibodies included: anti-human CD45 V500 (HI30), anti-human CD19 APC-H7 (HIB19), anti-human CD59 FITC (H19), anti-human CD92 BV605 (VIM15), anti-human CD267 BV421 (1A1-K21-M22) (all from BD Biosciences, USA), anti-human CD38 PE (REA572, Miltenyi Biotec, Germany), and anti-human CD320 AlexaFluor 647 (Antibodies Online, Germany). For CD32B, an anti-human unconjugated antibody (Thermo Fisher Scientific, Waltham, MA) was used with AlexaFluor647 conjugated secondary antibody. Cells were incubated with antibodies for 20 min at room temperature. After staining, the cells were washed and resuspended in 400 μL stain buffer before the acquisition on the BD FACSLyric™ flow cytometer (BD Biosciences, USA). Dead cells were excluded with 7-AAD staining (BD Biosciences, USA). Data were analyzed with FlowJo v10.2 software (BD Biosciences, USA). Gating strategy included sequential exclusion of doublets, dead cells, and non-PCs (CD45^+^/CD19^+^) prior to assessment of marker expression on CD38^+^ PCs. At least 50,000 events per sample were acquired. Percent positivity and mean fluorescence intensity (MFI) were quantified, and results were averaged across replicates.

## 3 Results

### 3.1 Identification of genes with increased expression in PCs

**Figure 1:**
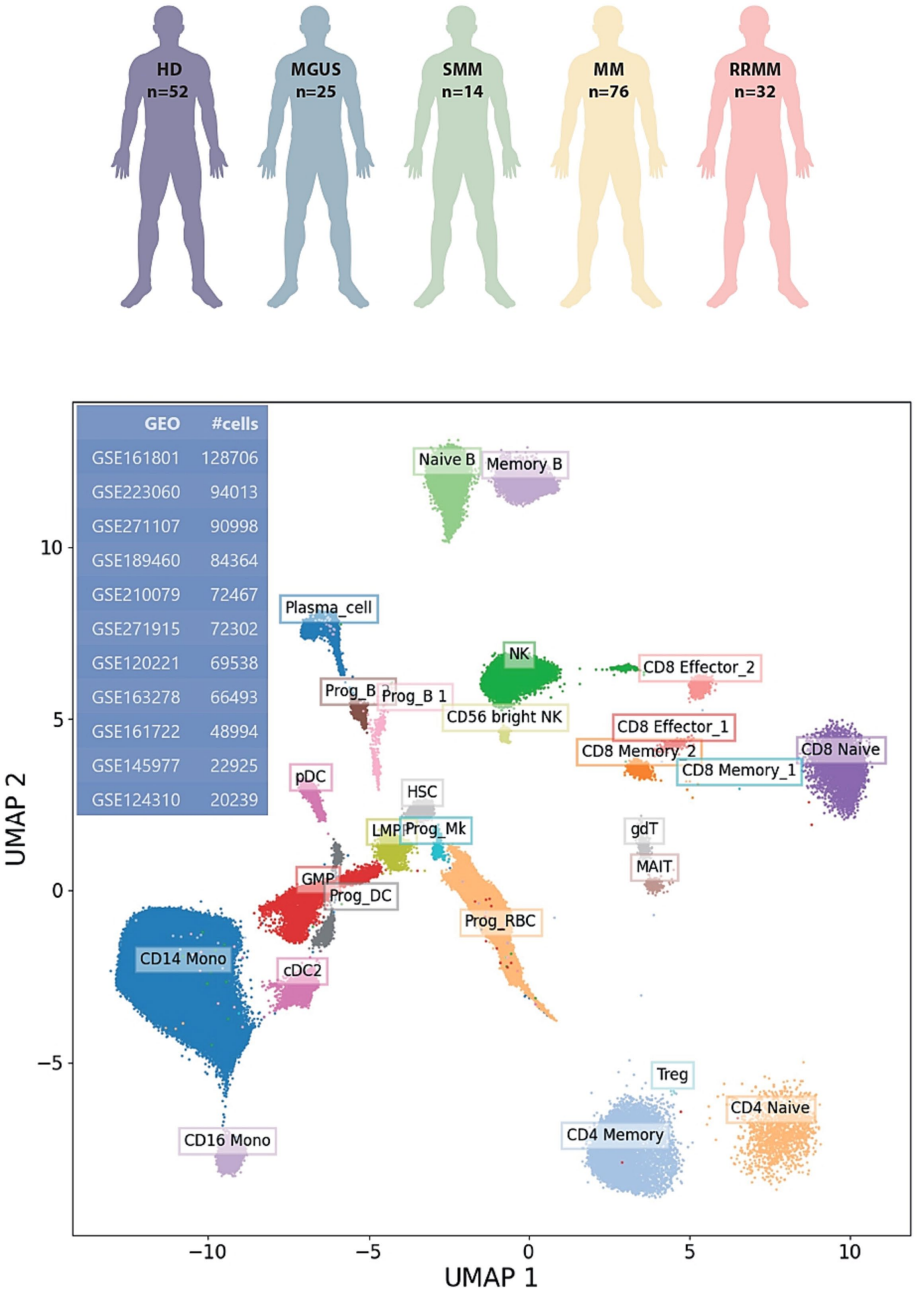
Uniform manifold approximation and projection (UMAP) plot created using R software, showing the clustering of individual cells from 11 publicly available datasets of the scRNA-seq analysis including HD, MGUS, SMM, and RRMM bone marrow (BM) samples. Each point which represents a single cell is colored according to annotated immune cell types. GEO, Gene Expression Omnibus; GSE, GEO Series.

**Figure 2:**
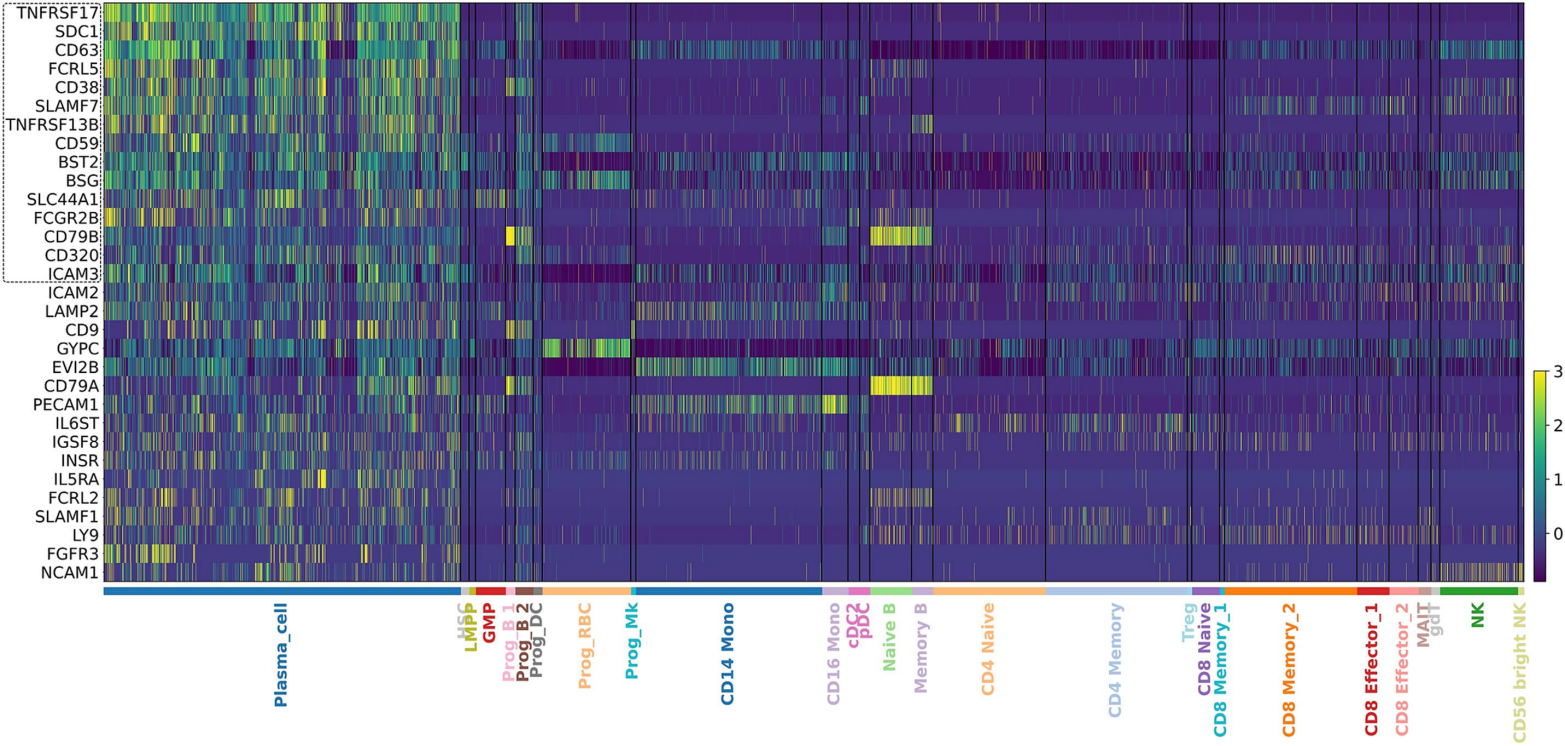
Heatmap depicting the expression levels of candidate plasma cell-specific marker genes across annotated immune cell types. Each row represents a gene, and each column corresponds to an immune cell type. The color intensity reflects the relative expression level, with higher expression indicated by darker shades. The inset highlights the identified 15 genes with increased expression in plasma cells (PCs) which encode for surface proteins.

**Figure 3:**
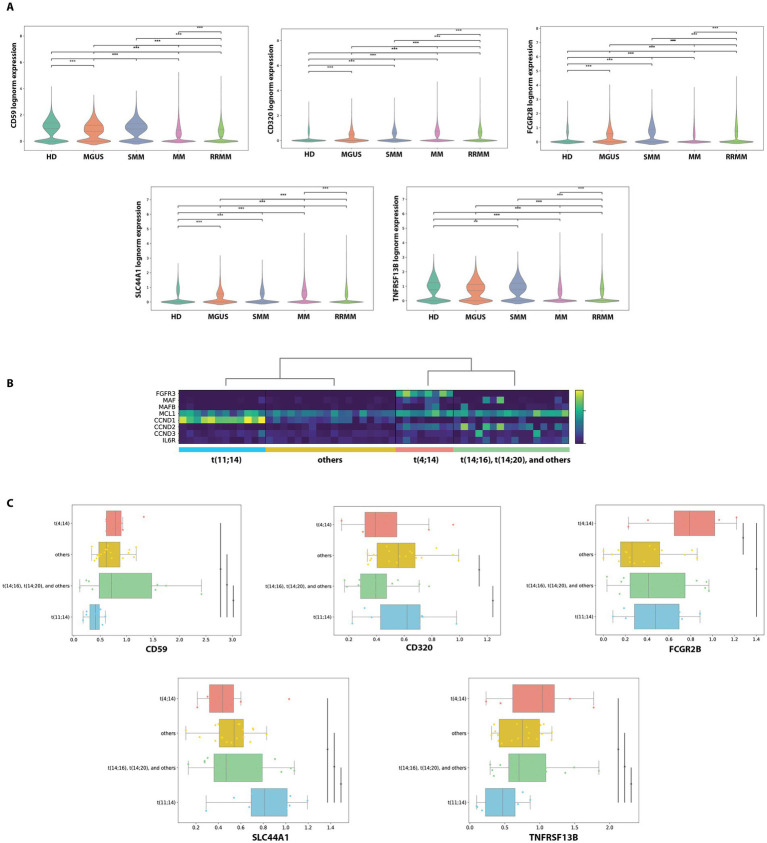
Gene expression and cytogenetic clusters in MM. **(A)** Violin plots showing gene expression levels for *TNFRSF13B*, *CD59*, *FCGR2B*, *SLC44A1*, and *CD320* in HD, MGUS, SMM, MM, RRMM. Statistical comparisons were conducted using the Kruskal–Wallis test for all pairwise contrasts. **(B)** Heatmap of the pseudobulk expression profiles showing normalized expression levels for eight putative proxy markers of cytogenetic aberrations across 54 MM patients. **(C)** Box plots illustrating the differential expression of *TNFRSF13B*, *CD59*, *FCGR2B*, *SLC44A1*, and *CD320* across the four cytogenetic patient clusters. Statistical significance was evaluated using two-sided *t*-tests for all pairwise comparisons *p* < 0.05 (*), *p* < 0.01 (**), *p* < 0.001 (***), *p* < 0.0001 (****).

We performed our integrated analysis from 11 datasets involving a total of 771,039 cells, of which 193,600 were identified as PCs (Figure 1). By comparing PCs transcriptome to the one of the whole remaining bone marrow cellularity, we found 15 genes with increased expression in PCs that encode for surface proteins: TNFRSF17 (BCMA), SDC1 (CD138), CD63, FCRL5 (FCRH5), CD38, SLAMF7 (CS1), TNFRSF13B (TACI), CD59, BST2, BSG, SLC44A1 (CTL1), FCGR2B, CD79B, CD320, ICAM3 (Figure 2). As expected, the list included several well-established therapeutic targets such as BCMA (TNFRSF17), CD138 (SDC1), CD38, SLAMF7, and FCRL5. Beyond these “canonical” antigens, our attention focused on five genes—TNFRSF13B (TACI), CD59, FCGR2B (CD32B), SLC44A1 (CD92), and CD320—that to date have received limited clinical exploration in the context of MM. We compared their log-normalized expression levels in plasma cells across different disease stages, namely healthy donors (HD), MGUS, SMM, MM, and RRMM (Figure 3A). For these five genes, there are significant differences in expression levels across the various conditions. When comparing expression across disease stages, distinct patterns emerged. CD320 and SLC44A1 displayed a progressive increase from MGUS through smoldering disease to overt and relapsed/refractory myeloma, suggesting a link with disease evolution. TNFRSF13B and FCGR2B were significantly higher in MM and RRMM compared to MGUS and healthy donors, while CD59 appeared particularly elevated in SMM and maintained high expression in advanced stages. These findings indicate that the five candidate molecules are not only enriched in malignant plasma cells but also differentially regulated during disease progression. To demonstrate that the molecules CD320, SLC44A1, TNFRSF13B, CD59, and FCGR2B exhibit restricted expression in PCs, we consulted the publicly available Human Protein Atlas database (40). CD320, FCGR2B, and TNFRSF13B showed a predominantly plasma cell–restricted protein expression, with only limited additional expression in vascular/endothelial compartments observed for CD320 and FCGR2B. In contrast, although SLC44A1 and CD59 were strongly expressed in plasma cells, their expression was not confined to this lineage (Supplementary Figure 2A). Consistently, analysis of cancer cell lines demonstrated a marked enrichment of expression in myeloma cell lines for all targets except CD59, which exhibited a broader distribution across cancer types (Supplementary Figure 2B).

### 3.2 Transcriptomic profiling identifies cytogenetic clusters in MM

To investigate whether the expression of these candidate genes was associated with known genetic subtypes of myeloma, we performed a clustering analysis of pseudobulk plasma cell transcriptomes derived from 54 newly diagnosed MM patients. This approach identified four molecular groups that closely reflected canonical cytogenetic categories: one cluster characterized by high CCND1 and MCL1 expression consistent with *t*(11;14), a second enriched in FGFR3 and MCL1 corresponding to *t*(4;14), a third driven by MAF and MAFB suggestive of *t*(14;16)/*t*(14;20), and a fourth group with heterogeneous profiles resembling hyperdiploid disease (Figure 3B). Notably, genes like FGFR3 and CCND1, which are directly involved in translocation events, emerged as strong transcriptional markers of their respective subtypes. The expression of MCL1, a survival gene, was elevated in multiple clusters, hinting at its broader role in disease biology. Further analysis showed that these cytogenetic clusters also significantly differ in the expression of TNFRSF13B, CD59, FCGR2B, SLC44A1, and CD320 (Figure 3C). The distribution and separation between groups indicate that these markers could be useful for identifying and classifying genetic subgroups of MM. This suggests a coordinated relationship between genetic alterations and cell surface phenotypes, which could have diagnostic and therapeutic implications.

### 3.3 Prognostic associations

**Figure 4:**
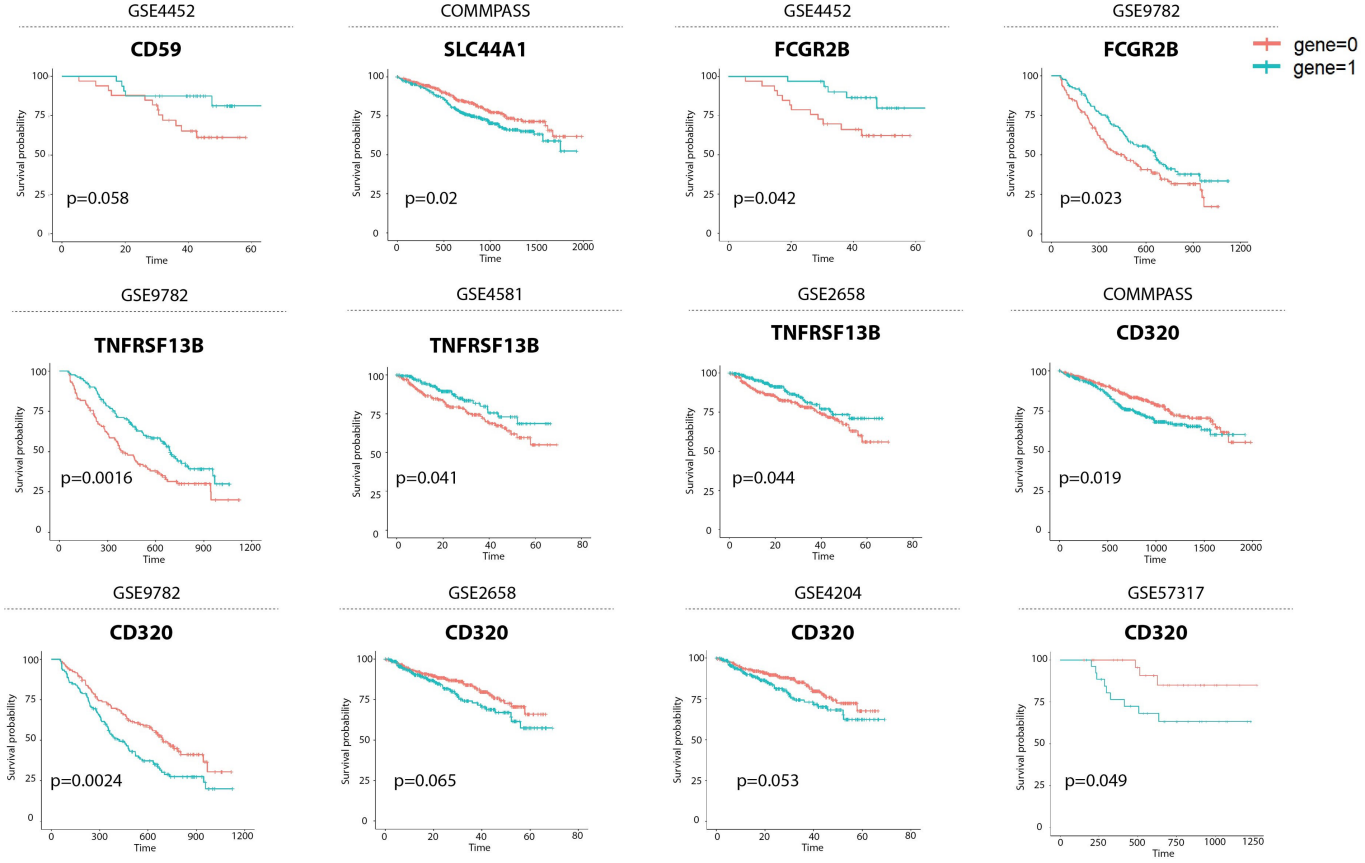
Kaplan–Meier survival curves showing the prognostic relevance of mRNA expression levels for CD59, SLC44A1, FCGR2B, TNFRSF13B, and CD320 genes. Cell populations with high expression of each gene are shown in blue, while populations with low expression of the same mRNA are shown in red. Reference datasets (GSE, GEO Series) are indicated at the top of each panel. Prognostic significance was assessed using the Cox proportional hazards model (95% confidence interval CI) and log-rank test, with *p* < 0.05 considered statistically significant.

To evaluate the clinical significance of the identified genes, we examined their association with patient outcomes using bulk transcriptomic datasets encompassing nearly 2,000 MM cases. Survival analyses demonstrated that differential expression of these molecules carried prognostic value. In particular, higher levels of TNFRSF13B, CD59, and FCGR2B correlated with improved survival, while elevated expression of CD320 and SLC44A1 was consistently linked to inferior prognosis. Among these, TNFRSF13B emerged as the strongest positive prognostic marker, whereas CD320 was the most robust predictor of poor outcomes (Figure 4). These observations underscore the relevance of the identified targets not only as potential therapeutic antigens but also as biomarkers for patient stratification.

### 3.4 Validation of scRNA-seq data through flow cytometry analysis

**Figure 5:**
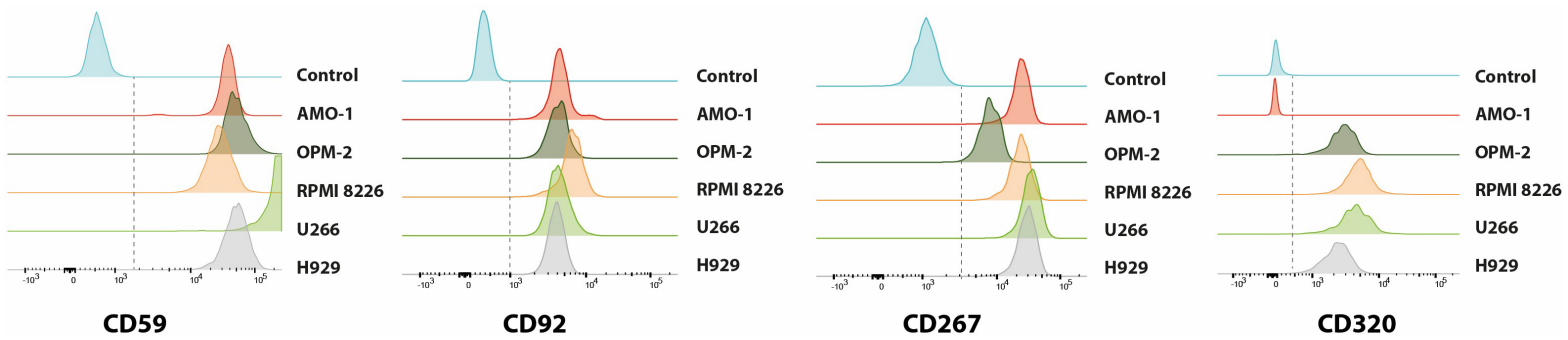
Histogram plots showing the cell surface expression of CD59, CD92, CD267, and CD320 molecules on different MM cell lines, including AMO-1, OPM-2, RPMI8226, U-266, and H929 as measured by flow cytometry analysis using fluorochrome-conjugated antibodies in triplicate experiments. Each panel represents the distribution of expression levels for the indicated marker, allowing comparison of protein abundance across the different cell lines compared to unstained control (in blue).

**Figure 6:**
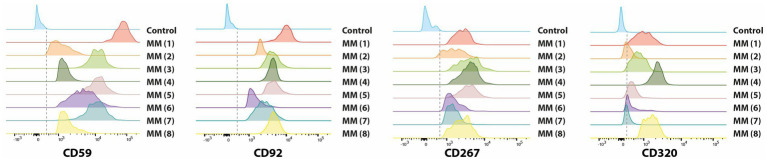
Histogram plots showing the cell surface expression of CD59, CD92, CD267, and CD320 molecules on malignant plasma cells (PCs) from eight bone marrow samples of newly diagnosed, untreated MM patients as measured by flow cytometry analysis using fluorochrome-conjugated antibodies in triplicate experiments. Each panel represents the distribution of expression levels for the indicated marker across the eight MM patients samples, allowing comparison of protein abundance compared to unstained control (in blue).

To experimentally validate our transcriptomic findings, we performed flow cytometry analysis on a panel of MM cell lines (AMO-1, U-266, OPM-2, RPMI-8226, H929) (Figure 5; Supplementary Figure 3) and on primary BM samples from eight newly diagnosed patients (Figure 6). The analysis confirmed surface expression of CD59, SLC44A1 (CD92), TNFRSF13B (CD267), and CD320 across most models, albeit with variable intensity. CD320 was particularly prominent in U-266 and OPM-2, detected at intermediate levels in RPMI-8226 and H929, and absent in AMO-1, highlighting inter-line heterogeneity. In contrast, CD32B expression was not reliably detected, as the signal was indistinguishable from secondary antibody controls (Supplementary Figure 4). In primary patient samples, malignant plasma cells exhibited strong positivity for CD59, CD92, CD267, and CD320, closely mirroring the transcriptomic data. Quantitative assessment indicated that the majority of PCs expressed at least one of these markers, supporting their potential as accessible and clinically relevant targets for immunotherapy (Supplementary Figures 5, 6).

## 4 Discussion

In this study we performed a comprehensive analysis of single-cell and bulk transcriptomic data to identify novel surface antigens that could serve as targets for CAR T-cell therapy in MM. By interrogating more than 770,000 cells from 11 scRNA-seq datasets and validating findings across ~2,000 patients from bulk expression cohorts, we provide robust evidence that CD320, SLC44A1, and TNFRSF13B, together with CD59 and FCGR2B, represent promising molecules for further exploration in immunotherapy. Our results highlight several important points. First, the candidate genes were consistently overexpressed in malignant plasma cells compared to healthy controls and showed stage-associated regulation across MGUS, SMM, MM, and RRMM. This suggests that these molecules not only reflect disease biology but may also contribute to the mechanisms driving progression. Notably, CD320 and SLC44A1 expression increased with advancing stage and were associated with inferior outcomes, whereas TNFRSF13B, CD59, and FCGR2B were linked to better prognosis. Such dual prognostic roles underscore the complexity of PC biology and the importance of contextualizing target selection within the clinical course of the disease. Second, the integration of cytogenetic subgroups with transcriptomic profiles revealed non-random associations between genetic lesions and surface antigen expression. CD320 enrichment in FGFR3/MCL1-driven myeloma and TNFRSF13B elevation in CCND1/MCL1-positive cases indicate that genetic background may shape the immunophenotypic landscape of malignant PCs. This connection between cytogenetic architecture and immunotherapy targets may guide patient stratification and improve the rational design of personalized treatments. Although we demonstrated associations between candidate gene expression and cytogenetic subgroups in patient samples, similar analyses were not performed in MM cell lines. Addressing this limitation in future studies will be essential to support functional and translational validation of these targets. Third, experimental validation by flow cytometry confirmed the presence of CD59, SLC44A1, TNFRSF13B, and CD320 on both cell lines and primary myeloma samples. Although CD32B was not reliably detected, the strong expression of the other four molecules provides proof-of-concept that these antigens are surface-accessible and therefore potentially actionable with antibody-drug conjugates-(ADCs), T-cell engagers-(TCEs), monoclonal antibody- or CAR-based strategies. The heterogeneity of CD320 expression across cell lines also suggests that its relevance may be subtype-dependent, an aspect that warrants further mechanistic studies.

Among these, CD320 stood out due to its distinctive expression profile, being significantly elevated in MM and RRMM compared to HD, MGUS, and SMM. CD320 encodes a receptor crucial for vitamin B12 absorption, which supports DNA synthesis and cell division. The CD320 gene encodes the transcobalamin receptor, which is expressed on the surface of many cell types and mediates the uptake of transcobalamin, a plasma protein conjugated with cobalamin (vitamin B12). The complex is internalized via receptor-mediated endocytosis. Overexpression of CD320 is associated with actively proliferating cells, while downregulation is linked to cellular quiescence. This physiological difference is explained by the need to ensure an adequate supply of vitamin B12 and folates during the early stages of the DNA synthesis cell cycle. In tumor cells (SMM, MM, and RRMM), CD320 is persistently overexpressed due to increased DNA synthesis and higher consumption of vitamin B12 and folates. Elevated CD320 expression in malignant PCs suggests that targeting this antigen with CAR T-cell therapy could reduce tumor cell proliferation while potentially minimizing toxicity, as it could provide specificity against neoplastic clones while minimizing side effects in healthy cells. The publicly accessible database of the Human Protein Atlas revealed that CD320 exhibited predominant expression in adipocytes and plasma cells, while TNFRS13B was primarily expressed in B-cells and plasma cells. TNFRSF13B, or TACI, a member of the TNF receptor superfamily, plays a key role in promoting myeloma cell growth and survival and contributes to resistance against conventional therapies. TACI interacts with APRIL and BAFF ligands, commonly overexpressed in the MM bone marrow microenvironment, activating downstream signaling pathways such as NF-κB and MAPK that enhance myeloma cell survival (41). Given its functional overlap with BCMA, TACI represents a promising target for new therapies, potentially complementing or enhancing BCMA-targeted treatments (42). Clinically, TACI expression is associated with a specific myeloma cell phenotype and poor prognosis in patients with low TACI expression, indicating its potential as a prognostic marker and its role in immune evasion within the bone marrow microenvironment. SLC44A1 encodes a transporter for choline, essential for acetylcholine production and membrane phospholipid synthesis. Overexpression of SLC44A1 in tumor cells may be related to increased mitotic activity and demand for phospholipids. Targeting SLC44A1 with CAR T-cell therapy could inhibit tumor growth by disrupting choline supply and phospholipid synthesis, offering a selective approach for treating cancer cells with high SLC44A1 expression. CD59, which inhibits complement membrane attack complex (MAC) formation, is often overexpressed in MM cells to evade immune destruction. Inhibiting CD59 could make tumor cells more susceptible to complement-mediated lysis, but this strategy requires precision to avoid damage to normal tissues. Additionally, CD59 could serve as a biomarker for identifying patients at higher risk of rapid disease progression or treatment resistance. FCGR2B, an inhibitory receptor that modulates immune responses by dampening cellular activation and antibody production, may be exploited by MM cells to evade immune surveillance. Its upregulation could prevent effective immune-mediated elimination of tumor cells.

Flow cytometry validation using AMO-1, U-266, OPM-2, RPMI8226 and H929 MM cell lines confirmed the expression of CD59, CD92, CD267 and CD320 antigens on malignant PCs, consistent with our scRNA-seq findings. The intermediate expression of CD320 in U-266 and OPM-2, compared to the absence in AMO-1, suggests variability in the expression of this marker among different MM cell lines. This could indicate that CD320 is not uniformly expressed in MM and may be associated with specific subtypes or states of differentiation within the disease. Prognostic analysis of approximately 2,000 MM patients demonstrated that all five genes are associated with varying prognostic outcomes. Specifically, higher levels of CD320 and SLC44A1 correlate with poorer prognosis, while TACI, CD59, and FCGR2B are linked to better outcomes. In summary, our findings identify CD320, SLC44A1, and TNFRSF13B as novel and clinically relevant surface antigens in MM. Their stage-associated expression, correlation with cytogenetic subtypes, and impact on prognosis position them as attractive candidates for next-generation CAR T-cell therapies.

Although CD320, SLC44A1, and TNFRSF13B emerged as promising candidates based on their expression patterns, regulation across disease stages, and association with survival outcomes, some aspects such as potential toxicity and therapeutic efficacy were not directly evaluated in this study and will require dedicated preclinical and clinical investigations. Future work should focus on mechanistic studies, preclinical models, and combinatorial approaches aimed at exploiting these targets to improve the depth and durability of responses in patients with MM.

## Data Availability

The original contributions presented in the study are included in the article/Supplementary material, further inquiries can be directed to the corresponding author/s.
