# Supplementary material for: Identification of CD320, SLC44A1 and TNFRSF13B as potential novel therapeutic targets for CAR T-cell therapy in multiple myeloma
Source: Front Med (Lausanne). 2026 Jan 13;12:1737919. doi: 10.3389/fmed.2025.1737919 (PMC12835215; doi:10.3389/fmed.2025.1737919)
Supplement: Supplementary file 1 [file Data_Sheet_1.docx]

**Supplementary Figure 1.**

Heatmap showing expression levels of candidate plasma cell-specific marker genes. After doublet removal, cells from different patients were integrated using Seurat’s anchoring-based approach. The gene expression matrix was normalized using the *sctransform* method, and the top 2000 variable genes were selected for principal component analysis. Dimensionality reduction was performed and unsupervised clustering was applied to identify cell populations. Plasma cells (PCs) were subsequently isolated and reclustered to explore heterogeneity. Subcluster annotation was performed based on canonical marker gene expression.

**
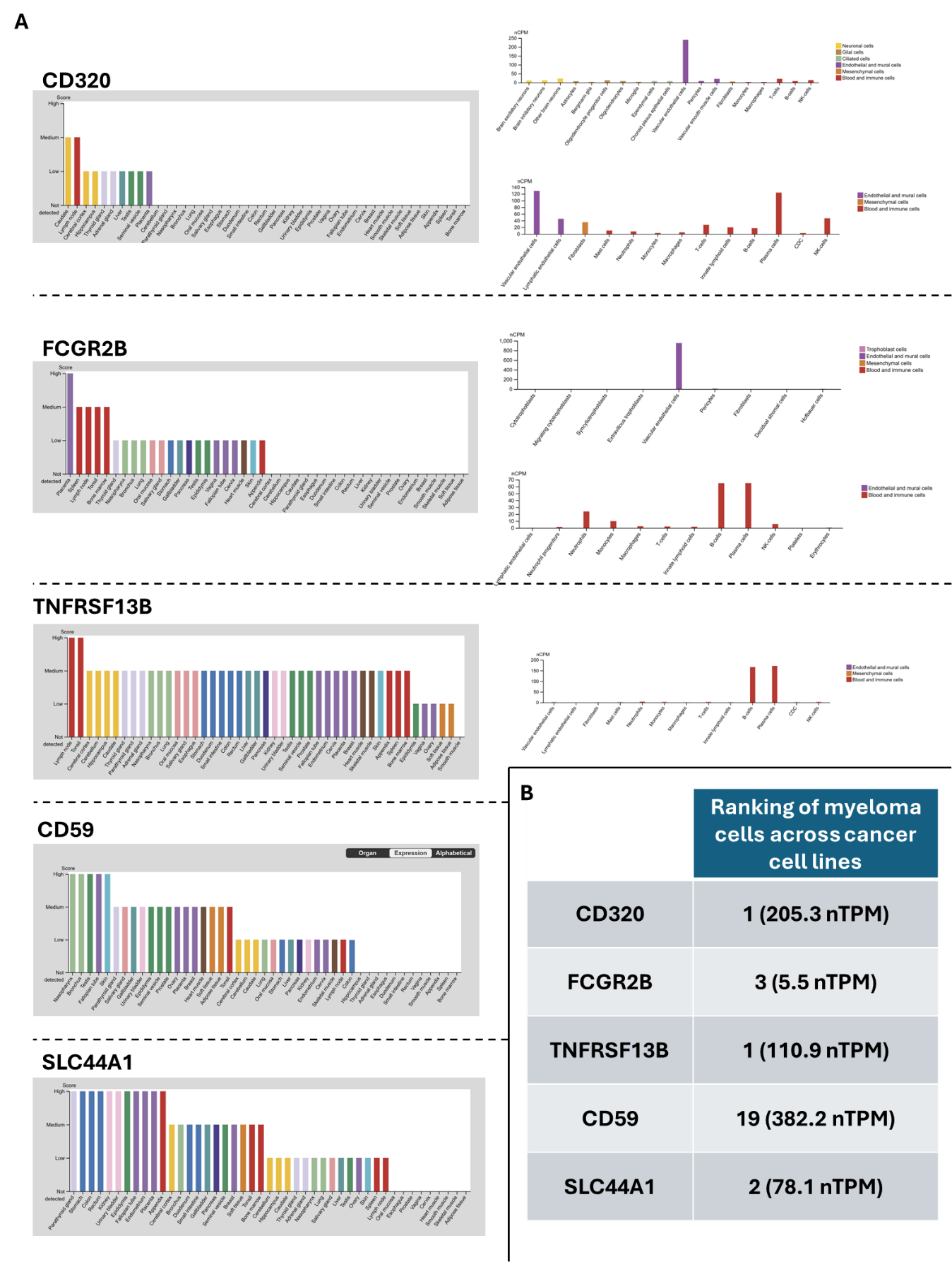
**

**Supplementary Figure 2.**

A: Bar charts depict protein expression levels across different tissues. Notably, for CD320, FCGR2B, and TNFRSF13B, tissue-level protein expression closely mirrored RNA expression, with plasma cells emerging as one of the most relevant contributing cell types. B: Gene expression analysis across all cancer cell lines included in the Protein Atlas revealed that, with the exception of CD59, which shows broad expression, myeloma cell lines rank among the most highly expressing cancers for the other candidate targets. nTPM: normalized protein-coding transcripts per million.


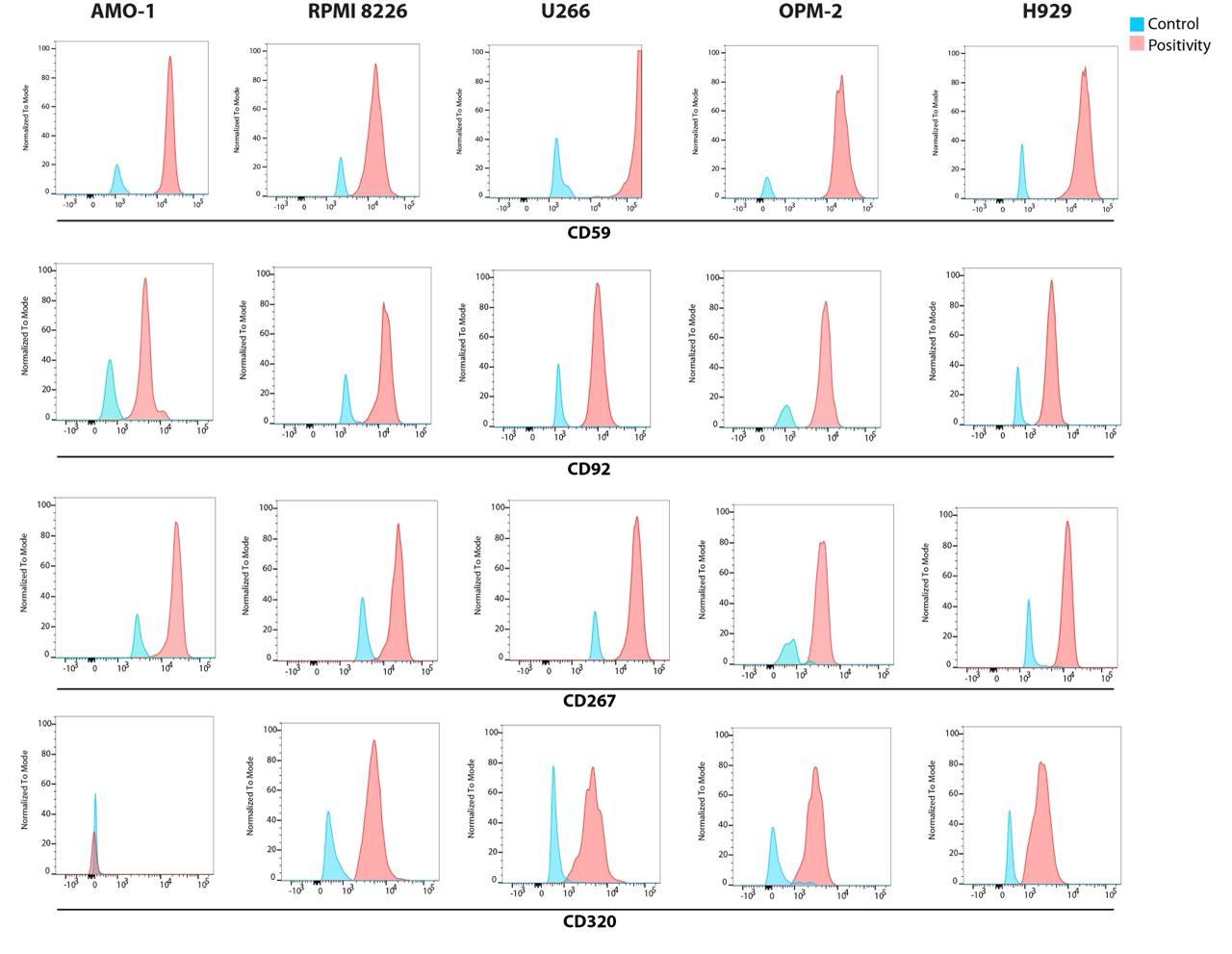


**Supplementary Figure 3.**

Histogram plots showing the cell surface expression of CD59, CD92, CD267, and CD320 molecules on different MM cell lines, including AMO-1, OPM-2, RPMI8226, U-266 and H929 as measured by flow cytometry analysis using fluorochrome-conjugated antibodies in triplicate experiments. Each panel represents the distribution of expression levels for the indicated marker, allowing comparison of protein abundance across the different cell lines compared to corresponding unstained controls (in blue).


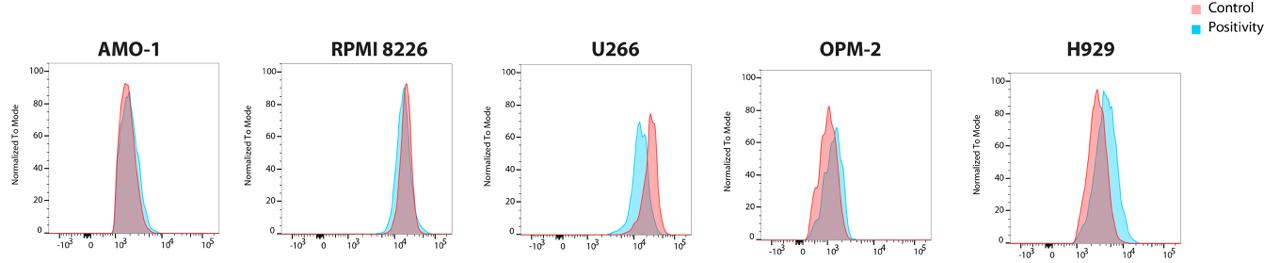


**Supplementary Figure 4.**

Histogram plots showing the cell surface expression of CD32B surface molecule (blue) on different MM cell lines, including AMO-1, OPM-2, RPMI8226, U-266 and H929 as measured by flow cytometry analysis in triplicate experiments. Each panel represents the distribution of expression levels for the indicated marker, allowing comparison of protein abundance across the different cell lines compared to the isotype controls (red).


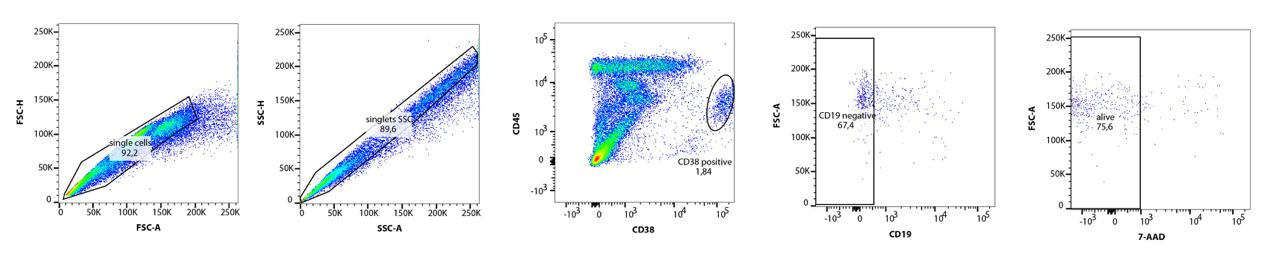


**Supplementary Figure 5.**

Dot plots showing the gating strategy used to identify malignant plasma cells from bone marrow samples of newly diagnosed, untreated multiple myeloma (MM) patients. Sequential gating steps include exclusion of doublets, followed by selection of plasma cells based on CD45, CD38, and CD19 expression.


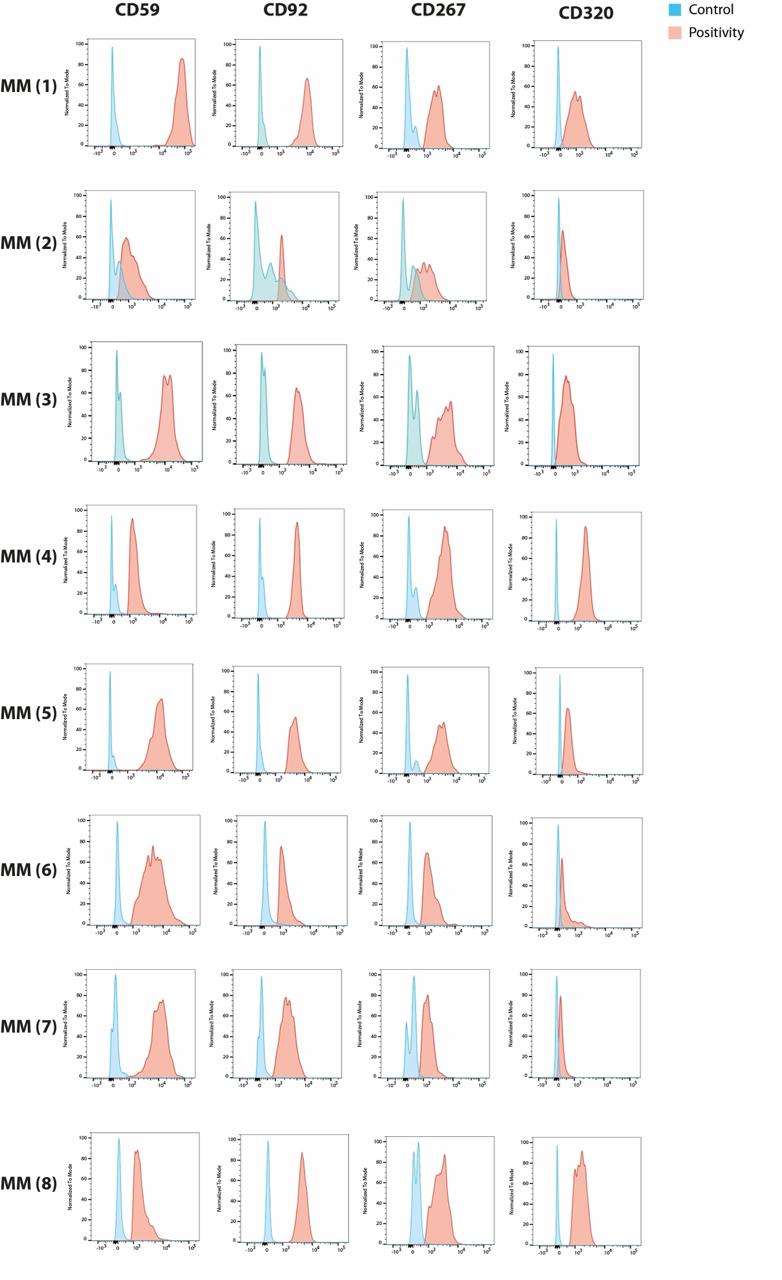


**Supplementary Figure 6.**

Histogram plots showing the cell surface expression of CD59, CD92, CD267, and CD320 molecules on malignant plasma cells (PCs) from eight bone marrow samples of newly diagnosed, untreated MM patients as measured by flow cytometry analysis using fluorochrome-conjugated antibodies in triplicate experiments. Each panel represents the distribution of expression levels for the indicated marker across the eight MM patients samples, allowing comparison of protein abundance compared to corresponding unstained controls (in blue).
